# Association of hysterectomy and invasive epithelial ovarian and tubal cancer: a cohort study within UKCTOCS

**DOI:** 10.1111/1471-0528.16943

**Published:** 2021-10-17

**Authors:** JA Taylor, M Burnell, A Ryana, C Karpinskyj, JK Kalsi, H Taylor, S Apostolidou, A Sharma, R Manchanda, R Woolas, S Campbell, M Parmar, N Singh, IJ Jacobs, U Menon, A Gentry-Maharaj

**Affiliations:** a MRC Clinical Trials Unit at UCL, Institute of Clinical Trials and Methodology, University College London, London, UK; b Department of Women’s Cancer, Institute for Women’s Health, University College London, London, UK; c Department Epidemiology and Public Health, Institute of Epidemiology and Health Care, UCL, London, UK; d Department of Surgery and Cancer, Imperial College Healthcare NHS Trust, London, UK; e Department of Obstetrics and Gynaecology, University Hospital of Wales, Cardiff, UK; f Barts Health NHS Trust and Wolfson Institute of Preventive Medicine, CRUK Barts Cancer Centre, Queen Mary University of London, London, UK; g Department of Gynaecological Oncology, Queen Alexandra Hospital, Portsmouth, UK; h Create Fertility, London, UK; i Department of Cellular Pathology, Barts Health NHS Trust, London, UK; j University of New South Wales, Sydney, NSW, Australia

**Keywords:** Hysterectomy, ovarian cancer, ovarian neoplasm, risk, type, UKCTOCS

## Abstract

**Objective:**

To investigate the association between hysterectomy with conservation of one or both adnexa and ovarian and tubal cancer.

**Design:**

Prospective cohort study.

**Setting:**

Thirteen NHS Trusts in England, Wales and Northern Ireland.

**Population:**

A total of 202 506 postmenopausal women recruited between 2001 and 2005 to the UK Collaborative Trial of Ovarian Cancer Screening (UKCTOCS) and followed up until 31 December 2014.

**Methods:**

Multiple sources (questionnaires, hospital notes, Hospital Episodes Statistics, national cancer/death registries, ultrasound reports) were used to obtain accurate data on hysterectomy (with conservation of one or both adnexa) and outcomes censored at bilateral oophorectomy, death, ovarian/tubal cancer diagnosis, loss to follow up or 31 December 2014. Cox proportional hazards regression models were used to assess the association.

**Main outcome measures:**

Invasive epithelial ovarian and tubal cancer (WHO 2014) on independent outcome review.

**Results:**

Hysterectomy with conservation of one or both adnexa was reported in 41 912 (20.7%; 41 912/202 506) women. Median follow up was 11.1 years (interquartile range 9.96–12.04), totalling >2.17 million woman-years. Among women who had undergone hysterectomy, 0.55% (231/41 912) were diagnosed with ovarian/tubal cancer, compared with 0.59% (945/160 594) of those with intact uterus. Multivariable analysis showed no evidence of an association between hysterectomy and invasive epithelial ovarian/tubal cancer (hazard ratio 0.98, 95% CI 0.85–1.13, *P* = 0.765).

**Conclusions:**

This large cohort study provides further independent validation that hysterectomy is not associated with alteration of invasive epithelial ovarian and tubal cancer risk. These data are important both for clinical counselling and for refining risk prediction models.

**Tweetable abstract:**

Hysterectomy does not alter risk of invasive epithelial ovarian and tubal cancer.

## Introduction

Hysterectomy with ovarian conservation is a common surgical procedure for benign indications.^1,2^ It has long been investigated as a risk factor for ovarian and tubal cancer (OC). The association was thought to be well established, with a 20–50% risk reduction for invasive epithelial OC being previously reported in women who underwent hysterectomy.^3–5^ The prevalent hypothesis was that hysterectomy prevents environmental carcinogens from ascending up the genital tract and damaging the ovaries. This protective effect was reported to differ by histological subtype, with the greatest risk reduction (43%) in clear-cell cancers.^6^

However, more recently, there have been conflicting reports on the association between hysterectomy and OC.^7,8^ A 2013 systematic review indicated a temporal shift with a 30% reduction in risk of OC in women diagnosed before 2000, and an 18% increase in risk in those diagnosed after 2000.^9^ The latter was confirmed by a 2014 cohort study of 51 052 postmenopausal women that reported a 36% increase in risk.^10^ A follow-up 2019 systematic review reported no association of hysterectomy and OC risk overall. A protective effect remained on subgroup analysis of invasive endometrioid/clear-cell cancers.^11^ More recently, an Australian study of 837 942 women has also reported no evidence of an association.^12^ The reasons for this discrepancy are probably related to incomplete data capture on removal of tubes and ovaries at the time of hysterectomy. This is especially relevant to data before 2000 when insights into the tubal origins of high-grade serous OC were lacking.

These conflicting reports emphasise the need for more studies with well-documented information on hysterectomy with conservation of adnexa and complete data on OC.^13,14^ Having clarity on this association is important both for risk prediction modelling as well as day-to-day patient counselling. Of note, some professional societies (American Cancer Society: https://www.cancer.org/cancer/ovarian-cancer/causes-risks-prevention/prevention.html) still cite hysterectomy as a protective factor.

The United Kingdom Collaborative Trial of Ovarian Cancer Screening (UKCTOCS) has complete self-reported data on hysterectomy from baseline, updated where possible from multiple sources, as well as complete independently confirmed OC diagnosis. We report on the association between hysterectomy (with conservation of one or both adnexa) and invasive epithelial OC risk in women who participated in the trial.

## Methods

### Study design

This is a cohort study within UKCTOCS, a multicentre randomised controlled trial of OC screening in the general population. In all, 1.2 million women were invited from Health Authority Registers adjoining 13 trial centres in England, Wales and Northern Ireland. Trial design has been described elsewhere.^15,16^ In brief, between 17 April 2001 and 29 September 2005, 202 638 postmenopausal women (aged 50–74 years) were recruited and randomised to no screening (control, *n =* 101 359), annual screening using CA125 interpreted using the Risk of Ovarian Cancer Algorithm (ROCA) with transvaginal ultrasound scan as a second-line test (multimodal screening, *n* = 50 640) or annual screening with transvaginal ultrasound (*n* = 50 639).

### Exposure (hysterectomy with conservation of one or both adnexa)

Study entry was recruitment (2001–05) when all participants completed a questionnaire where they documented if they had undergone a hysterectomy and separately whether they had both ovaries removed. Following this, information on hysterectomy was derived from multiple sources to ensure capture of as complete data as possible on the exposure variable on this large cohort over time. These included (1) self-reporting of hysterectomy (‘have you ever had a hysterectomy/removal of womb since joining the trial?’) including date on two postal follow-up questionnaires (3–5 years postrandomisation – FUQ1 and in April 2014 – FUQ2); (2) administrative data from Inpatient and Outpatient NHS Hospital Episode Statistics (HES, 1998–2014) for women recruited from England (the relevant HES data fields were searched using OPCS [Office of Population Censuses and Survey’s Classification of Surgical Operations and Procedures] codes for abdominal and vaginal hysterectomy (Q07.1–Q08.9)) (Table S1); (3) copies of surgical and pathology reports from hospital records that were retrieved for women who reported gynaecological surgery; (4) annual transvaginal scan data for the 48 230 eligible women from the ultrasound group (Table S2). All data sources with the exception of the ultrasound scan data were available for all the randomised women irrespective of group allocation for hysterectomy after randomisation (Table S2). However, for women who self-reported hysterectomy at baseline, it was only in one-quarter (48 230 women) that we had an additional data source, their baseline pelvic ultrasound scan. However, it needs to be noted that we have previously verified the high accuracy of self-reported hysterectomy in this cohort.^17^

As conservation of one or both ovaries and tubes was vital in the definition of exposure, oophorectomy status was similarly derived from medical notes, HES data (OPCS codes Q22.1–Q22.9 bilateral oophorectomy; Q23.1–23.9, unilateral oophorectomy; Q24.1–24.9 other excision of adnexa or uterus) or by self-reporting. Women with two separate notifications of unilateral oophorectomy on different dates were classified as having undergone bilateral salpingo-oophorectomy. Women self-reported if (and when) (‘have you had your ovaries removed?’ yes/no; right, left or both ovaries) since joining the trial on the two postal follow-up questionnaires (FUQ1 and FUQ2). It is assumed that if women had their ovaries removed that the fallopian tubes would have also been taken out at surgery.

The outcome for this study was invasive epithelial ovarian/tubal cancers defined by WHO 2014^18^ diagnosed by 31December 2014. Outcome was ascertained through (1) flagging for cancer registrations and deaths using NHS number through NHS Digital (England and Wales – till December 2016) and Northern Ireland (NI) Cancer Registry (till April 2015) and NI Health and Social Care Business Services Organisation (till August 2017); (2) linkage to National Cancer Intelligence Network (NCIN) data (till February 2015); (3) linkage to HES; (4) self-reporting in follow-up questionnaires; (5) direct communication from trial participants/their families; (6) trial centre reports. Copies of medical notes were retrieved for all women with a possible ovarian/tubal cancer (one of 19 pre-specified International Diseases Classification, tenth revision, codes), with final diagnosis and cancer site, Type (I, II or Uncertain)^19^ assigned by an independent outcomes review committee, as described previously.^15^ In view of the different outcomes in Type I (slow growing, indolent cancers including low-grade serous, endometrioid, clear-cell, mucinous) and in Type II (aggressive, mainly high-grade serous cancers accounting for most of the mortality), the outcomes committee assigned Type to each.

Potential confounding variables included body mass index (BMI) calculated as weight (kg)/height (m^2^), use of the oral contraceptive pill (OCP), parity (pregnancies lasting <6/>6 months), current hormone replacement therapy (HRT) use, history of tubal ligation, infertility (‘Have you ever had any treatment for infertility?’ Yes/No), personal history of cancer (including breast) and family history of ovarian and breast cancer collected at recruitment. Conventional covariate adjustment was used rather than propensity-score-based methods, as studies have shown that there is little difference in performance. In particular, certain propensity-score methods may give imprecise estimates^20^ and propensity-score matching can even increase imbalance and bias.^21^

Although hysterectomy was ascertained at the beginning of the study, as data on hysterectomy was captured from multiple sources throughout the long follow-up period, the exposure status was updated where appropriate. For participants who underwent hysterectomy following recruitment (study entry), follow-up time was split by date of hysterectomy. Hysterectomy was considered as a time-varying covariate with the time before hysterectomy classified as ‘unexposed’ and time after hysterectomy classified as ‘exposed’. For the women diagnosed with ovarian/tubal cancer, only hysterectomy performed at least 1 year before diagnosis date was included in the analysis. In a few women where date of hysterectomy was missing, information on how the derived dates of hysterectomy were calculated is presented in Appendix S1.

### Censorship data

Censorship for this analysis included bilateral oophorectomy, death from any cause, loss to follow up or 31 December 2014, whichever occurred first. In women diagnosed with ovarian/tubal cancer, date of diagnosis was used to derive follow-up time.

### Statistical methods

Cox proportional hazards regression was used, with age used as the time scale. Hence, although the effect of age cannot be directly estimated using a Cox model, its impact on OC is accounted for as part of unspecified baseline hazard function. Age at entry was calculated using the UKC-TOCS randomisation date, as hysterectomy status was recorded on the recruitment questionnaire.

Hazard ratio (HR) estimates for hysterectomy and all available a priori risk factors for ovarian and tubal cancer (tubal ligation, HRT use, OCP use, pregnancies longer than 6 months, family history of ovarian and breast cancer, BMI self-reported at study entry, age at last period, time since last period and age at first period) were performed. These variables were included individually in the Cox regression model to obtain univariate estimates of their hazard ratio relating to ovarian/tubal cancer risk overall and separately for Type I and Type II cancers.

All baseline variables (tubal ligation, HRT use, OCP use, pregnancies <6/>6 months, personal history of breast cancer and OC, family history of breast cancer and OC, BMI, age at last period, time since last period, and age at first period) were considered as confounders by analysing their association with hysterectomy status and OC risk separately with univariate analysis. HRT and OCP use were used instead of duration of use because of completeness of data. The final model included the known OC risk factors/a priori covariates tubal ligation, HRT use, OCP use, pregnancies over 6 months of gestation, family history of ovarian and breast cancer and BMI.

The multivariable analysis used Cox proportional hazards regression to estimate hazard ratio and corresponding 95% CI. When analysing the relationship by Type I or Type II, OC not in the association outcome were censored at date of diagnosis, rather than being classed as events.^22^ We further tested the proportional hazards test assumption that the test had not been violated to ensure that the Cox model was a valid statistical test for this analysis.

As HES data were only available for women residing in England, a sensitivity analysis restricted to those women was undertaken. All analyses were completed using STATA (version 14; StataCorp., College Station, TX, USA).

## Results

Of the 202 638 women randomised to the trial, 95 were excluded because they were identified as having a history of OC (*n* = 4), had both ovaries removed (*n* = 65), exited registry (*n* = 23) before randomisation, or withdrew consent (*n* = 3). In 37 women we had incomplete information regarding hysterectomy that they had self-reported during follow up. The final cohort therefore consisted of 202 506 women. Final adjustment in the multivariable model reduced this number to 199 556 women.

At study entry, the median age of the cohort was 60.6 years (interquartile range [IQR] 55.9–66.1 years). Median follow up from randomisation was 11.1 years (IQR 9.96–12.04 years). There was complete follow up until death, OC diagnosis or censorship date in 98.9% of women. Follow-up was incomplete in only 2253 (1.1%) women. Overall this amounted to over 2.17 million person-years of follow up. In total, 41 912 (20.7%) women underwent hysterectomy with conservation of one or both adnexa (Table 1); 32 899 (78.5%) women self-reported hysterectomy on the recruitment questionnaire and a further 9013 women underwent hysterectomy during follow up. A greater proportion of those who underwent hysterectomy had undergone tubal ligation, reported HRT use at recruitment (with longer duration of use), ever been pregnant, had higher BMI and were less likely to have received infertility treatment. Their age at the last period was lower. The extent of missing data was limited, ranging from 0.3% (for pregnancies >6 months) to 1.3% (for pregnancies <6 months) (Table 1).

During follow up, 1176 (0.58%) women were diagnosed with invasive epithelial OC, of whom 178 were Type I (15.1%), 890 were Type II (75.7%) and 108 were Type Uncertain (9.2%). The majority of cancers were high-grade serous (720, 61.2%), with the remaining comprising low-grade serous cancers (39, 3.3%), serous (grade not known and designated Type Uncertain) (28, 2.4%), mucinous (35, 3.0%), clear cell (49, 4.2%), endometrioid (86, 7.3%), carcinosarcoma (51, 4.3%) and carcinoma not otherwise specified (168, 14.3%).

Univariate analysis demonstrated that 0.55% (231/41 912) of the women who had undergone hysterectomy were diagnosed with invasive epithelial OC, compared with 0.59% (945/160 594) of those with an intact uterus, with a crude hazard ratio of 0.98 (95% CI 0.85–1.14) (Table 2). Reduction in invasive epithelial OC risk was noted in the crude associations for tubal ligation, ever use of OCP and parity (in Type I cancers), with an increase in risk for HRT use and family history of ovarian and breast cancer.

The final cohort with complete data included 199 556 women. However, the number of observations was higher (203 368), reflecting the splitting of time period at exposure into two observations in women who had a hysterectomy after recruitment. After adjusting for tubal ligation, HRT use, OCP use, pregnancies >6 months, BMI and family history of ovarian and breast cancer, the hazard ratio for invasive epithelial OC in women who had hysterectomy with conservation of at least one ovary compared with those who did not was 0.96 (95% CI 0.83–1.11, *P* = 0.578) (Table 3, Model 1). The multivariable association did not differ by Type (after adjusting for the above confounders), with a hazard ratio of 1.08 (95% CI 0.74–1.57; *P* = 0.691) for Type I and 0.96 (95% CI 0.81–1.13; *P* = 0.606) for Type II invasive epithelial OC (Table 3, Models 2 and 3). The proportional hazards test confirmed that the assumption had not been violated (χ^2^ = 1.69, *P =* 0.989), and therefore the Cox model was a valid statistical test for this analysis.

A sensitivity analysis restricted to women residing in England (where completeness of hysterectomy could be additionally confirmed through HES) demonstrated an adjusted hazard ratio of 0.97 (95% CI 0.82–1.15; *P* = 0.721).

## Discussion

### Main findings

In this large prospective cohort of 202 506 participants with well-annotated data, we found no evidence of an association between hysterectomy with conservation of one or both adnexa and invasive epithelial OC. Our effect estimates were unchanged when analysis was limited to women with hospital administrative data that provided additional confirmation of hysterectomy during follow up. This null effect persisted for both Type I and Type II OC.

Our findings and those of more recent studies suggest that the previously accepted protective effect between hysterectomy with ovary conservation and OC (Table S3) is not reliable. This has important implications for clinical decision-making in premenopausal women undergoing hysterectomy for benign indications, particularly in the age group 45–50 years. Patient information on OC in the UK continues to indicate that although hysterectomy has been considered as a potential protective factor for OC, that this association is currently considered uncertain.^23^ It is important that the growing evidence is shared with women to enable them to make a better informed decision.

### Interpretation

Our results of a null association are in keeping with recent reports from the Australian study^12^ and the European Prospective Investigation into Cancer and Nutrition (EPIC) cohort.^24^ The former was a population-based record linkage study of 837 942 Australian women for whom data over a 27-year period were available from electoral, hospital, births, deaths and cancer records. Data on hysterectomy with dates were available from hospital records and a cancer registry provided data on OC diagnosis. The study showed no decrease in risk for OC overall or serous subtype and although there was a trend towards a decrease in risk for mucinous, endometrioid and clear-cell cancers, this was not statistically significant. There was, however, a significant decrease in OC risk in women with endometriosis or fibroids (HR 0.17, 95% CI 0.12–0.24, and HR 0.27, 95% CI 0.20–0.36, respectively) regardless of subtype.^12^ The EPIC cohort included 334 126 women followed up until 2010 who had data on reproductive and hormone-related risk factors with hysterectomy ascertained at baseline using a standardised questionnaire. The data on OC (histology, grade and invasiveness) was available from cancer registries and pathology record review. EPIC showed a null effect with a non-statistically significant decrease in risk of clear-cell cancers.^12^

Our findings differ from earlier studies that reported an association. It is important to note that our focus was invasive epithelial OC whereas some case–control studies included benign ovarian tumours.^5^ Moreover, many varying definitions of OC were used.^3,5,10,24^ Invasive epithelial OC in our study was independently reviewed by an Outcomes Committee with site assigned as per the WHO 2014 classification, which included tubal cancers, the majority of which were previously assigned as primary peritoneal. The inconsistency between earlier studies and more recent data could also be influenced by the inclusion of women with no tubes or ovaries. The Nurses’ Health Study (NHSI and NHSII),^25^ which reported a protective effect (hazard ratio 0.80, 95% CI 0.49–0.90) had self-reported data on hysterectomy and oophorectomy at study entry (1992–95) but no further updates during follow up. Decreased use of HRT (which increases OC risk) in women after hysterectomy following publication of the initial Women’s Health Initiative results^26^ could have further contributed to this effect.

The lack of an effect of hysterectomy on Type I/II subgroup analysis was also noted in the EPIC cohort^24^ and a previous case–control study.^27^ The OC3 consortium metaanalysis of 19 prospective cohort studies (5584 cases) found a protective effect that was limited to clear-cell cancer (relative risk 0.57, 95% CI 0.36–0.88).^6^ This was also noted in the 2019 systematic review (incorporating the OC3 data), which reported a null association with OC overall but a protective effect for endometrioid/clear cell-cancers.^11^ In our study the latter cancers were grouped as Type I together with low-grade serous and mucinous cancers. It is likely that any effect on clear-cell cancers, if present, was masked by the small numbers (*n* = 49).

Recent evidence suggesting a tubal origin of OC^19^ has led to a change in surgical practice with tubes being increasingly removed during hysterectomy with conservation of ovaries. There is already evidence from retrospective studies that this is associated with a decreased risk of invasive epithelial OC.^28^ Currently large prospective studies are underway to estimate more accurate effect size.

The effect for all other known OC risk factors in our study was in line with the literature with a decreased risk associated with OCP, parity and tubal ligation and an increased risk with HRT, family history of OC and higher BMI. Risk stratification based on genetic and epidemiological data is increasingly used to predict a woman’s lifetime risk of developing OC.^29,30^ Risk models described so far have included OCP, parity, endometriosis, tubal ligation and family history of OC^31^ and more recently BMI, age at menopause and unilateral oophorectomy. Current efforts have focused on using prospective cohorts^32^ to build such models. Providing clarity on hysterectomy with ovary conservation as a risk factor for OC will aid these efforts.

### Strength and limitations

The major strengths of this study are the prospective cohort design, sample size and complete follow up through national registries (98.9% of participants) totalling >2.17 million person-years.^15^ Furthermore, all OC diagnoses were based on the reference standard of independent outcome review. Complete data on hysterectomy with conservation of at least one ovary beyond recruitment was ensured through linkage to electronic hospital administrative records. UKCTOCS eligibility criteria ensured that women had at least one intact ovary and were censored when both adnexae were removed. Combining multiple data sources improved the definition of both case and exposure.^33^ The availability of data on OC risk factors allowed us to adjust for most known covariates, unlike in the recent Australian study.^12^

Limitations of the study include the possibility of some bias in women who self-reported hysterectomy and removal of one or both ovaries. However, we have previously reported on the validity of self-reported hysterectomy compared with transvaginal ultrasound scan in women with intact ovaries in this cohort.^17^ We have assumed that where women have reported conservation of ovaries at hysterectomy this has included conservation of tubes as well, based on routine practice in the NHS during that period. We were unable to adjust for some risk factors, such as endometriosis.^34^ Previous data suggests a significantly reduced OC risk in women who underwent hysterectomy but had been previously diagnosed with endometriosis (HR 0.17, 95% CI 0.12−0.24) or fibroids (HR 0.27, 95% CI 0.20–0.36) compared with those without an OC diagnosis, or oophorectomy or hysterectomy for malignancy.^12^ We used BMI at recruitment. Unpublished data from a sub-study in our cohort suggests that BMI changes very little over time (0.44 kg gain between recruitment and 5–8 years post-recruitment). We could not explore the reported temporal change in association between women diagnosed with OC before 2000 (reduction in risk) and after 2000 (increase in risk)^9^ because recruitment in our trial started in April 2001. Furthermore, lack of data on date of hysterectomy at baseline limited our ability to assess exposure time for women who had undergone the procedure before trial entry.

## Conclusion and implications

Our prospective cohort study further confirms the lack of association between hysterectomy with conservation of one or both adnexa and invasive epithelial OC. Clarity on this association is important to ensure that premenopausal women undergoing hysterectomy for benign indications are able to make an informed decision about ovarian conservation. It is also relevant to OC risk prediction models, which are being developed for implementation of OC prevention strategies.

## Supplementary Material

Appendix S1

Supplementary Tables

## Figures and Tables

**Table 1:** Description of the cohort and distribution of hysterectomy status over each variable

Variable				Missingness *n* (%)	
	All women	Hysterectomy	No hysterectomy	Hysterectomy No	Hysterectomy
Overall cohort	202 506 (100)	41 912 (20.7)	160 594 (79.3)		
UKCTOCS group: Control	101 277 (50.01)	20 762 (49.5)	80 515 (50.1)	0(0)	0 (0)
UKCTOCS group: Multimodal	50 613 (24.99)	10 584 (25.3)	40 029 (24.9)	0 (0)	0 (0)
UKCTOCS group: Ultrasound	50 616 (24.99)	10 566 (25.2)	40 050 (24.9)	0 (0)	0 (0)
Tubal ligation	43 100 (21.3)	10 914 (26.0)	32 186 (20.0)	0 (0)	0 (0)
Use of HRT at recruitment	37 984 (18.8)	11 364 (27.1)	26 620 (16.6)	0 (0)	0 (0)
Ever use of OCP	120 669 (59.6)	24 801 (59.1)	95 868 (59.7)	0 (0)	0 (0)
Pregnancies <6 months: 0	137 941 (68.1)	27 606 (65.9)	110 335 (68.7)	551 (1.3)	2101 (1.3)
Pregnancies <6 months: 1	41 645 (20.6)	9120 (21.8)	32 525 (20.3)		
Pregnancies <6 months: 2+	20 268 (10)	4635 (11.1)	15 633 (9.7)		
Pregnancies >6 months: 0	23 482 (11.6)	3096 (7.4)	20 386 (12.7)	106 (0.2)	488 (0.3)
Pregnancies >6 months: 1	24 295 (12.0)	4196 (10.0)	20 099 (12.5)		
Pregnancies >6 months: 2+	154 135 (76.1)	34 514 (82.4)	119 621 (74.5)		
Ethnic origin: White	195 156 (96.9)	40 350 (96.2)	154 806 (96.4)	241 (0.6)	802 (0.5)
Ethnic origin: Other	6307 (3.1)	1321 (3.2)	4986 (3.1)		
Personal history of breast cancer	2562 (1.3)	500 (1.2)	2062 (1.3)	0 (0)	0 (0)
Family history of ovarian cancer	9177 (4.5)	1958 (4.7)	7219 (4.5)	0 (0)	0 (0)
Family history of breast cancer	44 983 (22.2)	9619 (22.9)	35 364 (22.0)	0 (0)	0 (0)
Infertility treatment	6627 (3.3)	1119 (2.7)	5508 (3.4)	0 (0)	0 (0)
Continuous variables, median (IQR)					
Duration of OCP use in those	5 (2−10)	5 (2−10)	5 (2−10)		
who had used it (years)					
Duration of HRT use for users at	8.11 (4.5−12.0)	10.2 (5.8−13.9)	7.3 (4.1−10.9)		
randomisation (years)					
BMI (kg/m^2^)	25.7 (23.3−29.1)	26.3 (23.7−29.7)	25.6 (23.2−29.0)		
Age at last period (years)	49.9 (45.9−52.6)	42.7 (38.2−47.4)	50.7 (48.2−53.2)		
Time since last period at	11.35 (5.29−18.47)	18.55 (13.07−24.12)	9.66 (4.32−16.13)		
randomisation (years)					
Age at randomisation (years)	60.56 (55.9−66.1)	61.00 (56.1−66.3)	60.45 (55.9−66.1)		
Age at first period (years)	13 (12−14)	13 (12−14)	13 (12−14)		

(%), % of participants in each variable group.

Values are given as number (percentage) or as median (IQR).

**Table 2:** Crude rate ratio for the univariate association between each variable and invasive epithelial ovarian/tubal cancer risk, overall and by Type I/II

Variable	Overall	All invasive epithelial ovarian/tubal cancer			Type I**			Type II**		
		n	HR (95% Cl)	P value	n	HR (95% Cl)	P value	n	HR (95% Cl)	P value
Total	*n (%)*	1176 (100)			178 (15.1)			890 (75.7)		
Hysterectomy										
Yes	41 912 (20.7)	231 (0.55)	**0.98 (0.85−1.14)**	0.819	39 (0.09)	**1.13 (0.79−1.62)**	0.493	173 (0.41)	**0.91 (0.77−1.07)**	0.252
No	160 594 (79.3)	945 (0.59)			139 (0.09)			717 (0.45)		
Tubal ligation										
Yes	43 100 (21.3)	199 (0.46)	0.78 (0.67−0.91)	0.002	27 (0.06)	0.66 (0.44−0.99)	0.047	151 (0.35)	0.78 (0.65−0.93)	0.006
No	159 406 (78.7)	977 (0.61)			151 (0.09)			739 (0.46)		
Use of HRT at recruitment										
Yes	37984 (18.8)	248 (0.65)	1.23 (1.07−1.42)	0.004	42 (0.11)	1.28 (0.90−1.81)	0.173	189 (0.50)	1.24 (1.05−1.46)	0.01
No	164522 (81.2)	928 (0.56)			136 (0.08)			701 (0.43)		
Ever use of OCP										
Yes	120 669 (59.6)	579 (0.48)	0.74 (0.66−0.84)	<0.0001	94 (0.08)	0.72 (0.52−0.98)	0.038	438 (0.36)	0.74 (0.64−0.85)	<0.0001
No	81 837 (40.4)	597 (0.73)			84 (0.10)			452 (0.55)		
Pregnancies <6 months										
0	137 941 (68.1)	831 (0.60)	ref	−	129 (0.09)	ref	−	619 (0.45)	ref	−
1+	61 913	327 (0.52)	0.87 (0.75−1.01)	0.085	37 (0.07)	0.79 (0.57−1.11)	0.176	256 (0.41)	0.93 (0.81−1.09)	0.398
Pregnancies >6 months										
0	23 482 (11.6)	153 (0.65)	ref	−	33 (0.14)	ref	−	110 (0.47)	ref	−
1+	178 430	1016 (0.57)	0.85 (0.72−1.01)	0.06	142 (0.08)	0.67 (0.39−0.83)	0.003	776 (0.43)	0.90 (0.74−1.10)	0.306
Family history of ovarian cancer										
Yes	9177 (4.5)	79 (0.86)	1.53 (1.22−1.92)	<0.0001		1.13 (0.58−2.22)	0.714		1.65 (1.28−2.13)	<0.0001
No	193 329 (95.5)	1097 (0.57)								
Family history of breast cancer										
Yes	44 983 (22.2)	288 (0.64)	1.14 (1.00−1.31)	0.049	36 (0.08)	0.89 (0.62−1.29)	0.539	220 (0.49)	1.15 (0.99−1.35)	0.062
No	157 523 (77.8)	888 (0.56)			142 (0.09)			670 (0.43)		
Infertility treatment										
Yes	6627 (3.3)	36 (0.54)	1.04 (0.75−1.46)	0.799		1.2 (0.56−2.57)	0.633		1.07 (0.74−1.57)	0.709
No	195 879 (96.7)	1140 (0.58)								
Quantitative variables										
OCP use (years)*	5 (2−10)***	4 (2_9)***	0.96 (0.95−0.97)	<0.0001	6 (2−10)***	0.99 (0.96−1.02)	0.421	5 (2_9)***	0.96 (0.94−0.97)	<0.0001
Duration of HRT	8.11 (4.5−12.0)***	9.89 (5.22−12.93)***	1.00 (1.00−1.00)	0.002	9.58 (5.9−12.1)***	1.03 (0.97−1.08)	0.325	9.62 (4.9−12.9)***	1.01 (0.98−1.03)	0.516
use for users at randomisation (years)										
BMI (kg/m^2^)	25.7 (23.3−29.1)***	25.6 (23.4−29.0)***	0.99 (0.98−1.01)	0.412	27.0 (23.7−30.7)***	1.03(1.01−1.06)	0.017	25.4 (23.2−28.6)***	0.90(0.97−1.00)	0.038
Time since last period at randomisation (years)	11.35 (5.29−18.47)***	13.4 (6.33−19.8)***	1.02 (0.97−1.08)	0.409	11 (5−18)***	0.90 (0.78−1.03)	0.128	11 (5−18)***	1.05 (0.99−1.11)	0.124

(%), % of ovarian/tubal cancer cases in each variable group.

Bold denotes the crude association of hysterectomy and ovarian cancer risk.

* Includes non-users.

** Ovarian/tubal cancer diagnoses of uncertain type *n* = 108 (9.2%).

*** Median (IQR) for women with ovarian/tubal cancer diagnosis.

**** Unreported because of the small number of events.

**Table 3:** Model 1 to Model 3: multivariable models for the association between hysterectomy and invasive epithelial ovarian/tubal cancer risk overall, by Type I and by Type II (*n* = 199 556; Observations = 203 368)

Adjusted model	Model 1 Invasive ovarian/tubal cancer overall (*n* = 1153)			Model 2 Type I Invasive ovarian/tubal cancer (*n* = 171)			Model 3 Type II Invasive ovarian/tubal cancer (*n* = 876)		
	HR	95% CI	P value	HR	95% CI	value	HR	95% CI	P value
Hysterectomy	0.96	0.83−1.11	0.58	1.08	0.74−1.57	0.691	0.96	0.81−1.13	0.606
Tubal ligation	0.81	0.69−0.95	0.008	0.67	0.44−1.03	0.07	0.81	0.68−0.97	0.021
HRT use	1.27	1.09−1.47	0.001	1.33	0.92−1.92	0.128	1.26	1.07−1.49	0.006
OCP use	0.74	0.66−0.84	<0.0001	0.74	0.54−1.03	0.072	0.74	0.64−0.85	<0.0001
Pregnancy >6 months	0.93	0.78−1.10	0.389	0.58	0.40−0.86	0.007	0.99	0.81−1.22	0.953
Ovarian cancer	1.54	1.22−1.94	<0.0001	1.03	0.51−2.10	0.928	1.68	1.30−2.17	<0.0001
family history									
Breast cancer	1.14	0.99−1.30	0.07	0.91	0.63−1.32	0.611	1.14	0.98−1.33	0.088
family history									
BMI	1	0.98−1.01	0.548	1.04	1.01−1.06	0.009	0.99	0.97−1.00	0.053

BMI, Body Mass Index; HR, Hazard Ratio; HRT, Hormone Replacement Therapy; IQR, Interquartile Range; OCP, Oral Contraceptive Pill.

## Data Availability

The data that support the findings of this study are available from the corresponding author upon reasonable request.
