# Supplementary material for: Association of hysterectomy and invasive epithelial ovarian and tubal cancer: a cohort study within UKCTOCS
Source: BJOG. Author manuscript; Available in PMC 2023 Dec 20. (PMC7615389; doi:10.1111/1471-0528.16943)
Supplement: Appendix S1 [file EMS190773-supplement-Appendix_S1.docx]

**Appendix S1**

*Date of hysterectomy*

Of all the methods of exposure ascertainment, the notification and date from the surgery and histopathology notes was considered the most accurate, followed by the HES data, follow-up questionnaire 1, follow-up questionnaire 2 and then the notification and derived dates for recruitment questionnaire and ultrasound scan date. As date of hysterectomy was not available if reported on the recruitment questionnaire or at the ultrasound scan, a hysterectomy date was derived from available data. Where recruitment questionnaire was the source of hysterectomy, the date the questionnaire completion was used as known exposure (hysterectomy) date. Where ultrasound scan was the source, the date was taken as the middle date between the last scan where both hysterectomy had not been noted and there was an endometrial thickness measurement, and the scan where hysterectomy was noted and the endometrial thickness was null or zero. If hysterectomy had been self-reported on follow-up questionnaires 1 or 2 but a date of the operation was not provided, questionnaire completion date was taken as the derived hysterectomy date. Further details on certainty of having undergone hysterectomy and date used are provided as Supplementary table 2.
