# Supplementary material for: Association of hysterectomy and invasive epithelial ovarian and tubal cancer: a cohort study within UKCTOCS
Source: BJOG. Author manuscript; Available in PMC 2023 Dec 20. (PMC7615389; doi:10.1111/1471-0528.16943)
Supplement: Supplementary Tables [file EMS190773-supplement-Supplementary_Tables.docx]

**Table S1: OPCS procedure codes for hysterectomy (HES data)**

| **OPCS Hysterectomy Codes** | **Procedure Description** |
| --- | --- |
| Q07.1 | Abdominal hystereocolpectomy and excision of periuterine tissue |
| Q07.2 | Abdominal hysterectomy and excision of periuterine tissue NEC |
| Q07.3 | Abdominal hysterocolpectomy |
| Q07.4 | Total abdominal hysterectomy |
| Q07.5 | Subtotal abdominal hysterectomy |
| Q07.8 | Other specified |
| Q07.9 | Unspecified |
| Q08.1 | Vaginal hysterocolpectomy and excision of periuterine tissue |
| Q08.2 | Vaginal hysterectomy and excision of periuterine tissue NEC |
| Q08.3 | Vaginal hysterocolpectomy NEC |
| Q08.8 | Other specified |
| Q08.9 | Unspecified: Includes vaginal hysterectomy NEC |

*Footnote: OPCS = Office of Population Censuses and Surveys Classification of Surgical Operations and Procedures from Hospital Episode Statistic data (HES); NEC = NEC (Not Elsewhere Classified)*

**Table S2: Sources of exposure ascertainment**

| **Source** | **Type** | **Data** | **Exposed** | **Unexposed** | **Date of exposure** |
| --- | --- | --- | --- | --- | --- |
| Recruitment questionnaire | Self report | Question to complete: Have you ever had a hysterectomy (removal of the womb)? | Yes box ticked | No box ticked | Taken as date recruitment questionnaire completed. |
| Postal follow up questionnaire 1 | Self report | Question to complete: ‘Since joining UKCTOCS have you had any of the following operations?’ Hysterectomy section was filled in, the participant was classed as exposed from the date of hysterectomy given. · If women had ticked hysterectomy and had written that they were due the operation, they were not classified as ‘had hysterectomy’. | Hysterectomy section completed | Hysterectomy section not completed | Where date of hysterectomy was completed this was taken as date of exposure. Where left blank but rest of section filled in date questionnaire completed was taken as minimum known exposure date. |
| Postal follow up questionnaire 2 | Self report | Question to complete: Have you had any of the following procedures since joining UKCTOCS? Tick box for operation to remove your womb, then space for date and treatment details. | Hysterectomy box ticked and a word search in operation details confirmed hysterectomy or an incorrectly spelled equivalent.^a^ | Hysterectomy was not ticked OR Hysterectomy was ticked and operation details were 'hysteroscopy'. | Where date of hysterectomy was completed this was taken as date of exposure. Where left blank but rest of section filled in date questionnaire completed was taken as minimum known exposure date. |
| HES data | Hospital reported | Data linkage to the Hospital Episode Statistics (HES) operation procedure codes (OPCs) for abdominal hysterectomy, vaginal hysterectomy and salpingo-oophorectomy. Codes for BSO and USO were also searched for and duplicates were removed. | Code for abdominal or vaginal hysterectomy linked to the participant in the absence of a code for BSO. | Codes for hysterectomy were not linked to participants OR participants had a code for BSO on the same date (participant censored but not exposed). | Date of procedure obtained from data linkage to HES data. |
| Scan data from the ultrasound and multi modal groups | Trial data taken by health professionals | TV scan noted hysterectomy. Hysterectomy was noted during the scan, where there was no hysterectomy an endometrium measurement was taken. | Hysterectomy ticked and endometrial thickess (at this and subsequent scans) was 0mm or not entered.^b^ | Hysterectomy not ticked | Date between the scan where hysterectomy was first noted was taken with no ET and the previous scan. |
| Medical Records requested during the trial | Hospital or GP records with details entered onto the Trial Database | Hysterectomy noted on retrieval of medical records.  Surgery information was entered onto the database and the fields of ‘hysterectomy done’ and ‘hysterectomy previously removed’ were searchable. | Hysterectomy done or previously done retrieved in database search as a separate entry to BSO | No hysterectomy entry into database. | Surgery date was entered into the database. Where hysterectomy was noted as 'previously done' a minimum date was taken as the date of the procedure being entered into the database. |

*^a^Data were cleaned by individually checking operation details when hysterectomy was ticked but details did not match the text search terms; b Records were checked individually where hysterectomy was classified and then a subsequent scan implied no hysterectomy. Where there was conflicting evidence , if the number of scans with hysterectomy ticked was greater than the number with a ET measurement given, women were classified as ‘had hysterectomy’;*

*Footnote: HES = Hospital Episode Statistics , BSO = Bilateral Salpingo-Oophorectomy, GP = General Practitioner*

**Table S3: Literature Review**

| **Author, year** | **Effect reported** | **Study design &/ Name** | **Participant description** | **Study size (n)** | **Time data collected** | **Definition of ovarian cancer (OC)** | **Simple hysterectomy** | **Hysterectomy with USO** | **Hysterectomy (Overall)** | |  |
| --- | --- | --- | --- | --- | --- | --- | --- | --- | --- | --- | --- |
|  |  |  |  |  |  |  | **OR (95% CI)** | **OR (95% CI)** | **OR, HR*** | **95% CI*** |  |
| Green *et al,* 1997^[3]^ | Protective | Matched Case-control | Australian women aged 18-79 | 1679 | 1990-1993 | Primary epithelial OC (independent pathologist review) | *Not reported* | | 0·64 | 0·48-0·85 |  |
|  |  |  |  |  |  |  |  |  |  |  |  |
| Chiaffarino *et al,* 2005^[4]^^ | Protective, no association with USO | Case-control | Italian women aged 18-79 | 3442 | 1992-1999 | Invasive epithelial OC | 0·6 (0·4-0·9) | 0·6 (0·3-1·1) |  |  |  |
|  |  |  |  |  |  |  |  |  |  |  |  |
| Rice *et al,* 2012^[5]^ | Protective | Meta-analysis | Case-control, pooled case-control, cohorts | 15,423 cases | 1969-2011 | Invasive OC in BRCAI/II carriers, Invasive epithelial OC, borderline tumours, benign serous and mucinous tumours, OC | 0·62 (0·49-0·79) | 0·60 (0·47-0·78) | 0·74 | 0·65-0·84 |  |
|  |  |  |  |  |  |  |  |  |  |  |  |
| Jordan *et al,* 2013^[9]^^ | Protective (Diagnosis pre-2000) | Systematic Review with meta-analysis | 6 cohort, 15 case-control studies | 21 estimates from 20 studies | 1971-2005 | OC pre-2000 | *Not reported* | | 0·7 | 0·65-0·76 |  |
|  | Harmful (Diagnosis post-2000) |  |  |  |  | OC post-2000 |  |  | 1·18 | 1·06-1·31 |  |
|  | Protective (overall) |  |  |  |  | OC all diagnoses |  |  | 0.81 | 0.72-0.92 |  |
| Rice *et al*, 2013^[7]^^ | No evidence | New England matched case-control (NECC2-5) | Women from Boston, Eastern Massachusetts and the State of New Hampshire, USA | 2265 cases; 2333 controls | 1984-2008 | Serous, borderline | 1·35 (0·74-2·47) | 1·23 (0·91-1·64) | 1·09 | 0·83-1·42 |  |
|  |  |  |  |  |  | Serous, invasive | 1·24 (0·91-1·69) | 0·62 (0·40-0·98) |  |  |  |
|  |  |  |  |  |  | Mucinous, borderline | 0·78 (0·31-2·00) | 0·92 (0·28-3·06) |  |  |  |
|  |  |  |  |  |  | Mucinous, invasive | 0·34 (0·31-2·00) | 0·90 (0·27-2·99) |  |  |  |
|  |  |  |  |  |  | Endometrioid | 0·83 (0·46-1·47) | 0·63 (0·29-1·36) |  |  |  |
|  |  |  |  |  |  | Clear cell | 0·87 (0·20-1·66) | 0·20 (0·03-1·53) |  |  |  |
|  |  |  |  |  |  | Other | 1·17 (0·65-2·11) | 0·47 (0·17-1·34) |  |  |  |
| Merritt *et al*, 2013^[26]^ | No evidence Type I, Type II; G1 Endometrioid/Clear cell Protective | New England Case-Control | Women from Eastern Massachusetts or New Hampshire, USA, aged 18 and over | 1571 cases; 2100 controls | 1992-2008 | Type I | *Not reported* | | 0.71 | 0.45-1.13 |  |
|  |  |  |  |  |  | Type II |  |  | 1.16 | 0.89-1.51 |  |
|  |  |  |  |  |  | Serous/mucinous - Grade 1 |  |  | 0.69 | 0.34-1.38 |  |
|  |  |  |  |  |  | Serous/other undifferentiated - Grade 2/3 |  |  | 1.33 | 1.03-1.73 |  |
|  |  |  |  |  |  | Endometrioid - Grade 1 |  |  | 1.45 | 0.73-2.88 |  |
|  |  |  |  |  |  | Endometrioid - Grade 2/3 |  |  | 0.49 | 0.23-1.02 |  |
|  |  |  |  |  |  | Endometrioid/clear cell |  |  | 0.54 | 0.34-0.86 |  |
|  |  |  |  |  |  | Clear cell |  |  | 0.47 | 0.17-1.32 |  |
| Gaudet *et al,* 2014^[23]^ | Harmful | Cancer Prevention Study II Nutrition Cohort | USA, postmenopausal women | 66,802 | 1999-2009 | Self-reported 'ovarian cancer' | 1·36 (1·03-1·78) | Combined with BSO | 1·16 | 1·03-1·78 |  |
|  |  |  |  |  |  |  |  |  |  |  |  |
| Rice et al, 2014^[24]^ | Protective | Nurses Health Studies I & II Cohorts | US female nurses age 30-55 and 25-42 | 238, 130 | 1976-2011 | Self-reported 'epithelial ovarian cancer' | 0·8 (0·66-0·97) | 0·70 (0·53-0·91) | 0·8 | 0·66-0·97 |  |
|  |  |  |  |  |  |  |  |  | 0·7 | 0·49-1·02 |  |
| Wang *et al,* 2016^[8]^^ | No evidence | Meta analysis | 22 case controls | 40,609 cases; 368,452 controls | 1988-2016 | Epithelial ovarian cancer | *Not reported* | | 0·97 | 0·81-1·14 |  |
|  |  |  |  |  |  |  |  |  |  |  |  |
| Wentzensen *et al*, 2015^[6]^ | Protective in clear cell only | OC3 Cohort Consortium | Postmenopausal women | 1.3 million | 1980-2014 | Invasive epithelial OC | *Not reported* | | 0.96 | 0.89-1.03 |  |
|  |  |  |  |  |  | Endometrioid |  |  | 0.84 | 0.66-1.07 |  |
|  |  |  |  |  |  | Mucinous |  |  | 0.72 | 0.51-1.02 |  |
|  |  |  |  |  |  | Clear cell |  |  | 0.57 | 0.36-0.88 |  |
|  |  |  |  |  |  | Serous |  |  | 1.03 | 0.94-1.13 |  |
|  |  |  |  |  |  | Grade I |  |  | 0.87 | 0.53-1.43 |  |
|  |  |  |  |  |  | Grade II |  |  | 1.05 | 0.84-1.33 |  |
|  |  |  |  |  |  | Grade III |  |  | 1.01 | 0.89-1.14 |  |
|  |  |  |  |  |  | Unknown Grade |  |  | 1.04 | 0.87-1.25 |  |
| Fortner *et al,* 2015^[22]^ | No Evidence | EPIC | 23 Centres in 10 countries including United Kingdom | 334,126 | 1992-2010 | Borderline | *Not reported* | | 1.06 | 0.49-2.32 |  |
|  |  |  |  |  |  | Invasive epithelial OC |  |  | 0.87 | 0.69-1.10 |  |
|  |  |  |  |  |  | Type I |  |  | 0.79 | 0.40-1.55 |  |
|  |  |  |  |  |  | Type II |  |  | 0.85 | 0.58-1.25 |  |
|  |  |  |  |  |  | Serous |  |  | 1.00 | 0.74-1.34 |  |
|  |  |  |  |  |  | Mucinous |  |  | 0.92 | 0.37-2.32 |  |
|  |  |  |  |  |  | Endometrioid |  |  | 1.00 | 0.50-2.01 |  |
|  |  |  |  |  |  | Clear cell |  |  | 0.3 | 0.04-2.28 |  |
| Dixon-Suen *et al*, 2019 ^[11]^ | No evidence, protective for endometriosis indication | Retrospective cohort study | Western Australia | 837,942 | 1988-2015 | Invasive epithelial OC | *Not reported* | | 0.98 | 0.85-1.11 |  |
|  |  |  |  |  |  | Serous |  |  | 1.05 | 0.89-1.23 |  |
|  |  |  |  |  |  | Mucinous |  |  | 0.55 | 0.28-1.06 |  |
|  |  |  |  |  |  | Endometrioid |  |  | 0.69 | 0.41-1.18 |  |
|  |  |  |  |  |  | Clear cell |  |  | 0.56 | 0.27-1.16 |  |
| Taylor *et al*, 2021 | No evidence overall, Type I or Type II· | Prospective cohort study (within UKCTOCS) | UK general population | 202,506 | 2001-2014 | Invasive epithelial OC | *Not reported* | | 0.96 | 0.83-1.11 |  |
|  |  |  |  |  |  | Type I |  |  | 1.08 | 0.74-1.57 |  |
|  |  |  |  |  |  | Type II |  |  | 0.96 | 0.81-1.13 |  |

** Association measure in hysterectomy +/- USO , or pooled estimates for multiple study analyses*

*Footnote: OC = Ovarian Cancer, OR = Odds Ratio, HR = Hazard Ratio, CI = Confidence Interval*

*^ confirmed by histology*
